# Vitamin D receptor gene polymorphisms and susceptibility of hand osteoarthritis in Finnish women

**DOI:** 10.1186/ar1874

**Published:** 2005-12-30

**Authors:** Svetlana Solovieva, Ari Hirvonen, Päivi Siivola, Tapio Vehmas, Katariina Luoma, Hilkka Riihimäki, Päivi Leino-Arjas

**Affiliations:** 1 Finnish Institute of Occupational Health, Department of Epidemiology and Biostatistics, Helsinki, Finland; 2 Finnish Institute of Occupational Health, Department of Industrial Hygiene and Toxicology, Helsinki, Finland; 3 Finnish Institute of Occupational Health, Department of Occupational Medicine, Helsinki, Finland; 4 Department of Radiology, Peijas Hospital, Helsinki University Central Hospital, Vantaa, Finland

## Abstract

We examined whether polymorphisms of the vitamin D receptor (*VDR*) gene was associated with individual risk of hand osteoarthritis (OA). Radiographs of both hands of 295 dentists and of 248 teachers were examined and classified for the presence of OA using reference images. The *VDR Apa*I and *Taq*I genotypes were determined by PCR-based methods. No association was observed between the *VDR *polymorphisms and the odds of overall hand OA. However, the carriers of the *VDR t *allele or *At *haplotype were at almost half the odds of symmetrical hand OA (odds ratio [OR] = 0.60, 95% confidence interval [CI] = 0.38–0.94 and OR = 0.59, 95% CI = 0.38–0.93, respectively) compared with the carriers of the *T *allele and of the non-*At *haplotype, respectively. Increased odds of this disease, on the contrary, was observed for women with two copies of the *VDR a *allele (OR = 1.93, 95% CI = 1.99–3.70) compared with women with the *AA *genotype. Conversely, the *VDR a *allele carriage was associated with a tendency of lowered odds of osteophyte (OR = 0.51, 95% CI = 0.25–1.03). When the genotype data were used to construct haplotypes, the *VDR AaTt *joint genotype appeared to pose a remarkably lower odds (OR = 0.26, 95% CI = 0.08–0.91) of osteophyte compared with the *AAtt *joint genotype. As a novel finding we observed a joint effect of a low calcium intake and *VDR *polymorphisms on symmetrical OA; the OR was 2.64 (95% CI = 1.29–5.40) for carriers of the *aT *haplotype with low daily calcium intake compared with non-carriers of the haplotype with high daily calcium intake. Our results suggest that *VDR *gene polymorphisms play a role in the etiology of symmetrical hand OA. Moreover, the association between the *VDR *gene and OA may be modified by calcium intake.

## Introduction

Osteoarthritis (OA) is the most frequent cause of musculoskeletal disability in developed countries. Two main subsets of the disease are recognized: monoarticular OA and polyarticular OA [1]. Despite the fact that OA is the most common joint disease, its etiology remains unclear. Among the most commonly suspected risk factors are age [2], previous injury [3], and obesity [2,4]. Current evidence suggests a genetic component to OA, with the heritability for hand OA and knee OA ranging from 39% to 65% [5,6].

Some of the studies have proposed that genetic susceptibility may be more relevant to OA in women than to OA in men, and that the role of genes in the development and progression of OA may vary between joint groups [7].

Hand OA often aggregates with knee OA [5,8,9]. Epidemiologic studies have shown that women with radiographic changes in the knee or the hand have an increased bone mass after adjustment for age and other covariates as compared with those women without OA [10,11]. Genetic factors affecting bone density may therefore play a role in the development of OA.

The vitamin D endocrine system, consisting of the steroid vitamin D, the vitamin D receptor (VDR) and the metabolizing enzymes, plays an important role in skeletal metabolism, OA, the immune response, and cancer [12]. The *VDR *gene acts as an important regulator of calcium metabolism and bone cell function [13], and it was the first candidate gene to be studied in osteoporosis. Biological support for an association between the *VDR *genotype and OA comes from studies showing that serum levels of vitamin D are related to the progression of knee OA [14] and to incident changes of radiographic hip OA characterized by joint space narrowing [15].

It has been shown that common allelic variations in the *VDR *locus can be used to predict the bone turnover rate [16]. The polymorphisms at the 3' end of the gene include two polymorphisms detectable with *Bsm*I and *Apa*I restriction enzymes that are located in intron 8 and one polymorphism detectable with *Taq*I restriction enzyme located in exon 9, while a poly(A) microsatellite polymorphism is located in the 3' untranslated region of the gene. The *Apa*I, *Bsm*I, and *Taq*I polymorphisms have been shown to be in strong linkage disequilibrium [16].

Keen and colleagues [17] examined the *VDR Taq*I polymorphism, associated with bone mineral density and osteoporosis, in relation to OA among postmenopausal English women. They found that women carrying one or two *TaqI *wild-type alleles (*T*) had an approximately threefold risk of OA in the knee joints compared with homozygotes for the variant allele (*t*). Uitterlinden and colleagues [18,19], on the other hand, demonstrated that a *VDR aT *haplotype (the *T *allele together with variant *a *allele at the *Apa*I locus) was related to knee OA, especially in osteophyte formation among elderly Dutch men and women.

It can be expected that the same polymorphisms could be important for both knee OA and hand OA, although no association between the *VDR *genotypes and hand OA was found in Japanese women [20] and in American men and women [21]. Various factors may have contributed to these apparently discordant results among studies, including the differences in the *VDR *genotypes distribution, the genetic environment and age structure of the studied population [22], and, more specifically, failure to take into account factors that modulate the effect of *VDR *gene on the risk of OA. Observations that the *VDR *genotypes are associated with intestinal calcium absorption [23,24] and that calcium interacts with *VDR *gene polymorphisms in studies of bone density [25,26] may suggest that the effect of *VDR *polymorphisms on the risk of OA may be modified by daily calcium intake.

The Finnish population is genetically relatively homogeneous and represents an isolated gene pool, the isolation being caused by linguistic and geographic factors. To our knowledge, the association between the *VDR *gene polymorphisms and OA has not been studied in the Finnish population. The aim of the present study was to examine whether the *VDR *polymorphisms, which were previously associated with knee OA, are also associated with the risk of hand OA in Finnish middle-aged women. In addition, we evaluated whether the calcium intake modifies the relationship between the *VDR *gene polymorphisms and hand OA.

## Materials and methods

### Subjects

The potential study subjects were identified through the register of the Finnish Dental Association and the Finnish Teachers Trade Union. Four hundred and thirty six women aged 45–63 years were randomly selected from each occupational group using the place of residence as an inclusion criterion (Helsinki or its neighboring cities) for participation in a study concerning work-related factors and individual susceptibility in the etiology of hand OA. Of those who received the questionnaires in 2002, 295 (67.7%) dentists and 248 (56.9%) teachers participated in a clinical examination between October 2002 and March 2003. Participation in the study was voluntary and based on informed consent. The Hospital District of Helsinki and Uusimaa Ethics Committee for Research in Occupational Health and Safety approved the study proposal.

### Hand radiography and image analysis

Both hands of the participants were radiographed. Kodak X-ray films were exposed with Siemens X-ray equipment (48 kV, 10 mA, focus film distance = 115 cm; Siemens, Munich, Germany). The analogue radiographs were evaluated by an experienced radiologist (TV) who was blinded to the occupation, age, and all health data of the subjects.

Each distal interphalangeal (DIP) joint, proximal interphalangeal (PIP) joint, thumb interphalangeal joint, and metacarpophalangeal (MCP) joint of both hands was graded separately, and was classified for the presence of OA using a modified Kellgren and Lawrence system [27]. The classification criteria were: grade 0 = no OA, grade 1 = doubtful OA, grade 2 = mild OA, grade 3 = moderate OA, and grade 4 = severe OA [22]. Osteophytes were separately classified as absent (grade 0), minimal or questionable (grade 1), or distinct (grade 2). Reference images were used. The description of reference images has been given elsewhere [28].

The reliability of the readings was estimated by measuring intraobserver and interobserver agreements, using the weighted Cohen's kappa coefficient with quadratic weights [29], for 46 randomly chosen subjects. For this estimation, a second reading was independently performed by TV and another experienced radiologist (KL). The inter-observer agreement for OA ranged from 0.67 to 0.85 for DIP joints, from 0.39 to 0.61 for PIP joints, and from 0.18 to 0.69 for MCP joints. The intraobserver agreement for OA ranged from 0.73 to 0.88 for DIP joints, from 0.67 to 0.92 for PIP joints, and from 0.59 to 1.0 for MCP joints. The readings of TV only were used in the subsequent statistical analyses.

If the subject had at least three finger joints with radiographic OA of grade 2–4, she was classified as having finger OA. Symmetrical OA was defined as a subcategory of OA in at least two symmetrical pairs of the joints (if two joints of the hand are affected, the same joints of the opposite hand are also affected). If the subject had at least two finger joints with distinct osteophyte (grade 2), she was classified as having finger osteophyte.

### Genotyping analysis

All DNA samples were extracted from lymphocytes by a DNA extraction kit (PUREGENE^® ^DNA Purification Kit; Gentra Systems, Plymouth, MN, USA).

The *VDR Apa*I and *Taq*I genotypes were resolved by a PCR-based method employing primers described by Riggs and colleagues [30]. Briefly, the PCR reactions were set up as follows: 50–100 ng template, 1 U DNA polymerase (Biotools; B&M Labs, SA, Madrid, Spain), 0.2 mM dNTPs, 0.5 μM each primer and 1.5 mM MgCl_2 _in the magnesium-free PCR buffer (Biotools; B&M Labs, SA). After initial denaturation of 2 min at 94°C, 28 cycles of 10 s at 94°C, 20 s at 60°C, and 30 s at 72°C were performed, followed by a 5 min final extension at 72°C. Aliquots of the PCR products were digested with *Bsp*120I (Fermentas) and *Taq*I (Fermentas) for *Apa*I and *Taq*I polymorphisms, respectively, and were electrophoresed on 3% agarose gel containing ethidium bromide. The final results were interpreted from pictures of the gels photographed under UV light; alleles lacking restriction sites for *Apa*I and *Taq*I were denoted as *VDR A *and *T *alleles, and alleles with the restriction sites as *a *and *t*, respectively.

### Questionnaires

Data regarding individual characteristics were collected by a self-administered questionnaire that included items on anthropometric measures, use of hormone therapy, dietary habits, and smoking history.

The use of hormone therapy was assessed by the questions 'Do you use hormonal medication? If yes, what is the name of medication?' and 'How long have you been using this hormonal medication?'

Queries about dietary habits on a daily average basis included coffee consumption (the number of cups), milk/sour milk consumption (the number of cups, one cup = 2 dl), yogurt consumption (the number of deciliters), and cheese consumption (the number of slices). Alcohol consumption was reported on a weekly average basis (number of portions, one portion = 12 cl for wine, 0.33 l for beer, and 4 cl for vodka or liquor).

Daily calcium intakes from particular dairy products were calculated from a knowledge of amounts of calcium per 100 g (1 dl) of each dairy product and amount of each product consumed per day. Subjects were asked about their daily intake of calcium from vitamin supplements. Daily calcium intake was determined by adding up the calcium level of the dairy products and the amount of calcium from vitamin supplements. For the analysis, daily calcium intake was compared with Finnish limit values [31] and was classified into low intake (<400 mg/day), adequate intake (400–800 mg/day), and high intake (>800 mg/day).

Weight was measured without shoes to the accuracy of 0.1 kg. The body mass index (BMI) (weight [kg] per height squared [m^2^]) was calculated based on self-reported height and the weight measured during the clinical examination session. The BMI was placed into tertiles for logistic regression analysis (low, <22.5 kg/m^2^; medium, 22.5–25.5 kg/m^2^; and high, >25.5 kg/m^2^).

Based on their smoking history, subjects were classified into never daily smokers or daily (current or previous) smokers.

### Statistical analyses

Allele and genotype frequencies were compared between individuals with and without OA using Fisher's exact probability test or the chi-square test. Carriage rates for the alleles were calculated as the proportion of individuals with at least one copy of the allele. Each gene locus was also examined for an allele dosage effect, by comparing the numbers of individuals heterozygous and homozygous for the test allele among those with and without OA.

The *VDR *haplotypes were statistically reconstructed from population genotype data using the PHASE program with the Markov chain method for haplotype assignments [32].

A set of logistic regression analyses was performed to examine the association between the *VDR *genotypes and hand OA. To evaluate whether the association between the genotypes and OA is modified by daily calcium intake, the odds ratio (OR) of having OA was calculated as a function of daily calcium intake (low intake versus moderate or high intake), of the presence of a risk allele, and of their interaction. An absence of a risk allele and high or moderate daily calcium intake was used as reference group.

Crude and adjusted ORs and their 95% confidence intervals (CIs) were calculated using the SPSS statistical package (SPSS Inc., Chicago, IL, USA). The ORs were adjusted for the potential confounding factors of age, occupation, height, BMI, use of hormone therapy, daily calcium intake, and coffee and alcohol consumption. Since the crude and adjusted ORs did not differ significantly, only the adjusted ORs are shown.

## Results

The prevalence of the overall hand OA, symmetrical OA, and osteophytes among the female dentists and teachers aged 45–63 years were 29%, 21%, and 7%, respectively. The basic characteristics of the study subjects by their hand OA status (absence or presence of OA) are summarized in Table 1. Radiographic findings were more prevalent in the teachers, in the subjects who were older, and in more often users of hormone therapy.

The genotype and allele distributions of the *VDR Apa*I and *Taq*I loci did not deviate significantly from Hardy–Weinberg equilibrium, and the frequencies agreed with those previously observed in Finnish women [33].

Six of the possible nine joint genotypes were reconstructed by the PHASE program [26] from the *Apa*I and *Taq*I genotype data. Five of the genotypes (*AaTt*, *AaTT*, *aaTT*, *AATt*, and *AAtt*) were relatively common, exhibiting frequencies of 28.4%, 21.0%, 18.6%, 16.6%, and 11.8%, whereas the frequency for the *AATT *genotype was only 3.7%. The frequencies of the *VDR aT*, *At *and *AT *haplotypes were 43%, 34%, and 32%, respectively.

The age, height, BMI, occupation, use of hormone therapy, smoking history, coffee consumption, and alcohol drinking were similar in the *VDR *genotype groups, but the daily calcium intake was higher in the *tt *and *Tt *genotype groups compared with the *TT *genotype group (775 ± 382, 678 ± 380, and 647 ± 355 mg, respectively, *P *= 0.05). The proportion of ever-smokers was higher among the carriers of the *aT *haplotype compared with the non-carriers (24% versus 17%, *P *= 0.042).

No association was observed between individual *VDR *gene polymorphisms and the overall odds of hand OA. However, the *VDR aa *genotype posed an almost doubled odds of symmetrical hand OA (OR = 1.93, 95% CI = 1.00–3.70) compared with the *AA *genotype (Table 2). The women with at least one copy of the *VDR t *allele (Table 2) or carrying the *At *haplotype (Table 3) were at almost halved odds of this disease (OR = 0.60, 95% CI = 0.38–0.94 and OR = 0.59, 95% CI = 0.38–0.93, respectively) compared with the carriers of *T *allele and the non-*At *haplotype, respectively. On the other hand, the *VDR a *allele carriage was associated with a tendency (OR = 0.51, 95% CI = 0.25–1.03) to lowered odds of osteophyte.

The *VDR AaTt *joint genotype appeared to pose a remarkably lower odds of osteophyte (OR = 0.26, 95% CI = 0.08–0.91) compared with the *AAtt *joint genotype (Table 3).

The separate and joint effects of the *VDR aT *haplotype and a low calcium intake on the risk of hand OA are presented in Table 4. The presence of the *aT *haplotype alone was associated with an increased odds of symmetrical OA (OR = 1.58, 95% CI = 0.88–2.85) and with a decreased odds of osteophytes (OR = 0.39, 95% CI = 0.18–0.85). Similarly, there was no statistically significant association between a low calcium intake and OA among subjects without the *aT *haplotype. However, both factors (the *aT *haplotype and low calcium intake) acted synergistically to increase the odds of OA. We observed a joint effect of a low calcium intake and carriage of the *VDR aT *haplotype on symmetrical OA; the OR was 2.64 (95% CI = 1.29–5.40) for carriers of the *aT *haplotype with a low daily calcium intake compared with non-carriers of the haplotype with a moderate or high daily intake.

In agreement with previous observations [27] that the *VDR a *allele appeared to always be associated with the *T *allele, identical ORs for the *VDR a *allele carriage and the *aT *haplotype carriage were seen in the present study (Tables 2 and 3).

## Discussion

An age-dependent pattern for the presence of finger OA has been found among adult participants of the Tecumseh Community Health Study [34]. Among individuals under the age of 44 years OA was observed almost exclusively in the DIP joints, whereas among older participants the PIP and MCP joints were affected. Several studies provided clear evidence for a polyarticular subset of hand OA in women [28,35-37], with three major determinants of the pattern of polyarticular involvement being symmetry, clustering by row, and clustering by ray (in descending order of importance). While OA in a specific joint (monoarticular OA) may result from acute trauma [27] or from mechanical stress [38] to the joint, OA with multiple joint involvement (polyarticular OA) might be dominated by systemic factors to which all joints would be equally susceptible.

In the present study we aimed to examine more severe cases of OA, which are more likely to bear a genetic component. OA was therefore defined to be present if there was a radiograph reading of grade 2–4 in at least three joints of the fingers. OA was defined to be symmetrical if at least two symmetrical pairs of joints (the same joint in both hands) were affected.

Our findings suggest that there may indeed be a relationship between the *VDR Apa*I and *Taq*I polymorphisms and the risk of symmetrical hand OA in Finnish women. The *VDR aa *genotype, which has previously been associated with high bone mass [30], posed a nearly twofold increased odds of symmetrical hand OA as compared with the *AA *genotype associated with lower bone mass. In contrast, the odds of this disease was almost halved among those with the *VDR t *allele, which has previously been associated with a higher rate of bone turnover [39] and lower bone mass [40] compared with the *T *allele.

In our study, the association between the *VDR aT *haplotype and hand OA depended on the interaction with dietary calcium intake. A joint effect of a low calcium intake and carriage of the *VDR aT *haplotype on risk of symmetrical OA exceeded their additive effects, indicating that the *VDR aT *haplotype is a potential risk factor for OA among women with insufficient calcium intake. These relationships were independent of other risk factors such as age, occupation, BMI, use of hormone therapy, and smoking history.

Regulation of intracellular calcium plays a key role in hypertension, insulin resistance, and obesity [41]. A protective effect of dietary calcium in preventing bone fragility and certain cancers has been reported [42]. Several previous studies have recognized that the association between *VDR *alleles and bone mineral density may vary depending on the level of calcium intake [25,26,43-45], and there is evidence that calcium is able to enhance cartilage repair and stimulate collagen production [46]. The *VDR baT *haplotype has been related to enhanced abnormality in the calcium regulation of the parathyroid hormone secretion from adenomatous parathyroid cells of primary human parathyroid tumor [47]. However, no other study has evaluated the potential relation between calcium intake and *VDR *genotypes in OA etiology. Our finding, if confirmed, implies a considerable potential for a role of nutritional interventions for OA.

In the Framingham study [14] the growth of knee osteophytes was found to be associated with the vitamin D level. In the present study, women with the *VDR AaTt *joint genotype had the lowest odds and those with the *AATT *genotype the highest odds of hand osteophytes as compared with the *AAtt *genotype. The direction of these associations contrast the findings in the studies of osteophytes formation in the knee [17-19] and the lumbar spine [48], but agree with the findings related to the severity and presence of lumbar spine osteophytes [49].

The large amount of positive genetic association data in a number of diseases such as osteoporosis, OA, diabetes, and cancer [50] suggest functional consequences of *VDR *gene polymorphisms. The *ApaI *polymorphism is unlikely to have functional consequences, however, since it is located in intron 8 and is not affecting any splicing site or transcription factor binding site. Moreover, although the *Taq*I polymorphism is located in the coding sequence (exon 9) of the *VDR *gene, it has no effect to the encoded protein sequence [13]. Nevertheless, supporting the modifying role of the *VDR *polymorphisms in the VDR functionality, lower *VDR *mRNA levels were found to be associated with homozygosity for the *a *and *T *alleles [51].

Our study has several limitations. The cross-sectional study design precluded the assessment of an effect of dietary calcium intake on the incidence or progression of OA. The relatively small number of subjects reduced the power of the study to detect differences. Another limitation arises from the fact that calcium intake was assessed based on a questionnaire, and thus the recall bias may affect the accuracy of information gathered. Finally, vitamin D intake was not assessed in our population.

Despite these limitations, the present findings are not anticipated to be caused by selection bias. First, selection on the genotype is unlikely since all study subjects were of homogeneous Finnish origin. Second, the prevalence of hand OA among the women analyzed in this study is similar to that seen in other studies [52-54]. Third, the *VDR *genotype frequencies in this population did not significantly deviate from the Hardy–Weinberg equilibrium and the genotype frequencies were similar to those previously observed in Finnish women [33]. Neither could the associations be explained by other risk factors, since these potential confounders were controlled in all statistical analyses.

The lower level of interobserver agreement for radiographic findings was not surprising. Despite training and the use of reference images, each reader graded the radiographs according to his or her own inherent standard about what constituted a positive finding. The high intraobserver agreement suggests that the classification criteria applied here are highly reproducible. Because all radiographs were evaluated by one observer who was blind to subjects' genetic data, the high intraobserver repeatability implies that the comparison between subjects with different *VDR *genotypes was unbiased. The possibility of non-differential misclassification of osteoarthritis cannot be ruled out. Non-differential misclassification of a binary outcome usually produces bias toward the null.

It could be argued that at least some of the associations found in this study were spurious, considering multiple comparisons were performed. However, there are several arguments to support the consistency of these results rather than attributing them to chance. First, we hypothesized *a priori *that the interaction observed in this study would occur, based on the known biology of the *VDR *gene in regulation of calcium metabolism. Second, our sample was homogeneous in terms of age, gender, ethnicity, and genetic background. Third, haplotypes were constructed taking into account the knowledge that collective grouping of single nucleotide polymorphisms in haplotypes has a stronger association with the phenotype than individual polymorphisms. Finally, to minimize the number of analyses performed, the interaction hypothesis was tested with the risk haplotype only.

## Conclusion

Although the possibility remains that the studied polymorphisms do not directly affect the individual susceptibility of hand OA, but are in linkage disequilibrium with an unknown nearby susceptibility locus, our results provide evidence of the involvement of the *VDR *polymorphism in the etiology of symmetrical hand OA. In addition, our findings suggest a detrimental effect of low dietary calcium intake on OA. The findings remain to be weighted in future studies. Further studies into mechanisms underlying the relationships between calcium and OA may improve the understanding of the obtained results.

## Abbreviations

BMI = body mass index; CI = confidence interval; DIP = distal interphalangeal; MCP= metacarpophalangeal; PCR = polymerase chain reaction; PIP = proximal interphalangeal; OA = osteoarthritis; OR = odds ratio; VDR = vitamin D receptor.

## Competing interests

The authors declare that they have no competing interests.

## Authors' contributions

SS participated in the design of the study, performed the statistical analysis, and drafted the manuscript. AH and PS carried out the genotyping analysis. TV carried out the evaluation of hand radiographs. KL participated in the evaluation of reliability of radiographs' readings. HR conceived of the study and participated in its design. PLA conceived of the study and participated in its design and coordination. All authors read and approved the final manuscript.

## Figures and Tables

**Table 1:** Characteristics of the study population by their osteoarthritis (OA) status

OA category		*n *(%)	Age [mean ± SD] (years)*	Body mass index [mean ± SD] (kg/m^2^)	Daily calcium intake [mean ± SD] (mg)	Occupation^†^		Hormone therapy^‡^		Smoking status	
						
						Dentists (*n*)	Teachers (*n*)	Ever used (*n*)	Never used (*n*)	Ever smoked (*n*)	Never smoked (*n*)
Overall	No	383 (71%)	53.0 ± 5.2	24.3 ± 3.5	663.2 ± 356.3	223	160	187	196	88	297
	Yes	160 (29%)	56.3 ± 4.7	25.0 ± 3.8	707.7 ± 403.8	72 (24%)	88 (35%)	103 (35%)	57 (22%)	33 (28%)	127 (30%)
Symmetrical	No	431 (79%)	53.3 ± 5.2	24.3 ± 3.5	673.4 ± 363.2	245	186	216	215	99	332
	Yes	110 (21%)	56.5 ± 4.6	25.1 ± 3.8	688.1 ± 403.8	48 (16%)	62 (25%)	72 (25%)	38 (15%)	20 (17%)	90 (21%)
Osteophyte	No	504 (93%)	53.6 ± 5.2	24.4 ± 3.6	669.4 ± 371.0	272	232	264	240	113	391
	Yes	39 (7%)	57.9 ± 4.1	25.2 ± 3.5	765.3 ± 365.2	23 (8%)	16 (6%)	26 (9%)	13 (5%)	6 (5%)	33 (8%)

*P = 0.0001 for the comparison between hand OA status groups (Student's *t *test). ^†^*P *= 0.01 for the comparison between the persons with and without overall OA, and for the comparison between persons with and without symmetrical OA (Student's *t *test). ^‡^*P *= 0.004 for the comparison between the persons with and without overall OA, and for the comparison between persons with and without symmetrical OA (Student's *t *test).

**Table 2:** Association between the *VDR *genotypes and hand osteoarthritis (OA)

Genotype	Overall OA		Symmetrical OA		Osteophyte	
	
	Yes/no	Odds ratio (95% CI)^a^	Yes/no	Odds ratio (95% CI)^a^	Yes/no	Odds ratio (95% CI)^a^
*Apa*I^b^						
*AA*	51/123	1.0	29/145	1.0	18/156	1.0
*Aa*	77/191	0.98 (0.62–1.54)	56/212	1.43 (0.84–2.42)	14/254	0.46 (0.21–1.00)
*aa*	32/69	1.20 (0.67–2.15)	25/76	1.93 (1.00–3.70)	7/94	0.65 (0.24–1.75)
'*A*' allele carriage	128/303	0.82 (0.49–1.37)	85/357	0.65 (0.37–1.13)	32/410	1.01 (0.40–2.51)
'*a*' allele carriage	109/260	1.03 (0.67–1.59)	81/288	1.55 (0.94–2.56)	21/348	0.51 (0.25–1.03)
*Taq*I^c^						
*tt*	20/44	1.00	11/53	1.0	7/57	1.0
*Tt*	68/173	0.75 (0.39–1.44)	42/199	0.90 (0.41–1.97)	13/228	0.46 (0.16–1.26)
*TT*	71/164	0.92 (0.48–1.76)	57/178	1.53 (0.72–3.29)	19/216	0.73 (0.27–1.93)
'*t*' allele carriage	88/217	0.87 (0.58–1.30)	53/252	0.60 (0.38–0.94)	20/285	0.77 (0.39–1.53)
'*T*' allele carriage	139/337	0.83 (0.45–1.54)	99/377	1.19 (0.57–2.47)	32/444	0.58 (0.23–1.46)

^a^Odds ratio and 95% confidence interval (CI) adjusted for age (years), height (cm), occupation (1 = dentists, 2 = teachers [reference group]), hormone therapy (1 = ever used, 2 = never used [reference group]), current body mass index (1 = high, 2 = moderate, 3 = low [reference group]), daily calcium intake (1 = high [reference group], 2 = moderate, 3 = low), coffee consumption (number of cups per day), alcohol consumption (number of portion per week), and smoking history (1 = never smoked [reference group], 2 = ever smoked). ^b^A, absence of a restriction site; a, presence of a restriction site. ^c^T, absence of a restriction site; t, presence of a restriction site. For technical reasons, three samples were not genotyped for the *Taq*I VDR polymorphism.

**Table 3:** Association between the *VDR *haplotypes and hand osteoarthritis (OA)

Joint genotype^a^	Overall OA		Symmetrical OA		Osteophyte	
	
	Yes/no	Odds ratio (95% CI)^b^	Yes/no	Odds ratio (95% CI)^b^	Yes/no	Odds ratio (95% CI)^b^
*Aatt*	20/44	1.0	11/53	1.0	7/57	1.0
*AATt*	26/64	0.75 (0.35–1.61)	14/75	0.73 (0.29–1.83)	8/82	0.82 (0.2–2.56)
*AATT*	5/15	0.68 (0.20–2.38)	4/16	1.03 (0.26–4.08)	3/17	1.62 (0.33–7.92)
*AaTt*	43/111	0.75 (0.38–1.50)	28/125	0.99 (0.43–2.25)	5/149	0.26 (0.08–0.91)
*AaTT*	34/80	0.89 (0.43–1.84)	28/86	1.55 (0.67–3.54)	9/105	0.70 (0.23–2.13)
*AaTT*	32/69	1.00 (0.48–2.07)	25/76	1.63 (0.70–3.82)	7/94	0.61 (0.19–2.00)
*At *haplotype carriage	89/219	0.87 (0.58–1.30)	53/253	0.59 (0.38–0.93)	20/288	0.76 (0.38–1.52)
*AT *haplotype carriage	65/159	0.93 (0.62–1.41)	46/177	0.96 (0.61–1.52)	20/204	1.60 (0.78–3.27)
*aT *haplotype carriage	109/260	1.03 (0.67–1.59)	81/287	1.55 (0.94–2.56)	21/348	0.51 (0.25–1.03)
No *aT *haplotype^c^	51/123	1.0	29/144	1.0	18/156	1.0
One *aT *haplotype^d^	77/191	0.98 (0.62–1.54)	56/213	1.43 (0.84–2.42)	14/254	0.46 (0.21–1.00)
Two *aT *haplotypes^e^	32/69	1.20 (0.67–2.15)	25/76	1.93 (1.01–3.70)	7/94	0.65 (0.24–1.75)

^a^A, absence of a restriction site; a, presence of a restriction site; T, absence of a restriction site; t, presence of a restriction site. ^b^Odds ratio and 95% confidence interval (CI) adjusted for age (years), height (cm), occupation (1 = dentists, 2 = teachers [reference group]), hormone therapy (1 = ever used, 2 = never used [reference group]), current body mass index (1 = high, 2 = moderate, 3 = low [reference group]), daily calcium intake (1 = high [reference group], 2 = moderate, 3 = low), coffee consumption (number of cups per day), alcohol consumption (number of portion per week), and smoking history (1 = never smoked [reference group], 2 = ever smoked). ^c^No *aT *haplotype = *AATT *+ *AATt *+ *AAtt*.^d^One copy of the *aT *haplotype = *AaTT *+ *AaTt*. ^e^Two copies of the *aT *haplotype = *aaTT*.

**Table 4:** Combined effects of the *VDR *genotypes and daily calcium intake on odds of hand osteoarthritis (OA)

Carriage of the *aT *haplotype	Low calcium intake	*n*	Overall OA	Symmetrical OA	Osteophyte
No	No	131	1.0	1.0	1.0
No	Yes	43	1.02 (0.46–2.30)	1.73 (0.69–4.36)	0.28 (0.06–1.35)
Yes	No	269	0.92 (0.56–1.51)	1.58 (0.88–2.85)	0.39 (0.18–0.85)
Yes	Yes	100	1.58 (0.85–2.93)	2.64 (1.29–5.40)	0.52 (0.17–1.56)

Data presented as the odds ratio (95% confidence interval) adjusted for age (years), height (cm), occupation (1 = dentists, 2 = teachers [reference group]), hormone therapy (1 = ever used, 2 = never used [reference group]), current body mass index (1 = high, 2 = moderate, 3 = low [reference group]), coffee consumption (number of cups per day), alcohol consumption (number of portion per week), and smoking history (1 = never smoked [reference group], 2 = ever smoked).
